# Behavioral rhythms of an opportunistic predator living in anthropogenic landscapes

**DOI:** 10.1186/s40462-020-00205-x

**Published:** 2020-04-24

**Authors:** Yaiza Parra-Torres, Francisco Ramírez, Isabel Afán, Jacopo Aguzzi, Willem Bouten, Manuela G. Forero, Joan Navarro

**Affiliations:** 1 Institut de Ciències del Mar – CSIC, Barcelona, Spain; 2 Estación Biológica de Doñana – CSIC, Sevilla, Spain; 3 Theoretical and Computational Ecology, Institute for Biodiversity and Ecosystem Dynamics, University of Amsterdam, Amsterdam, The Netherlands

**Keywords:** Anthropogenic influence, Foraging ecology, Opportunistic seabirds, Rhythmic behavior, Tracking, Winning species

## Abstract

**Background:**

Human activities have profoundly altered the spatio-temporal availability of food resources. Yet, there is a clear lack of knowledge on how opportunistic species adapt to these new circumstances by scheduling their daily rhythms and adjust their foraging decisions to predicable patterns of anthropic food subsidies. Here, we used nearly continuous GPS tracking data to investigate the adaptability of daily foraging activity in an opportunistic predator, the yellow-legged gull (*Larus michahellis*), in response to human schedules.

**Methods:**

By using waveform analysis, we compared timing and magnitude of peaks in daily activity of different GPS-tracked individuals in eleven different habitat types, in relation to type of day (i.e., weekday vs. weekend).

**Results:**

Daily activity rhythms varied greatly depending on whether it was a weekday or weekend, thus suggesting that gulls’ activity peaks matched the routines of human activity in each habitat type. We observed for the first time two types of activity as modelled by waveforms analysis: marine habitats showed unimodal patterns with prolonged activity and terrestrial habitats showed bimodal patterns with two shorter and variable activity peaks.

**Conclusions:**

Our results suggest that gulls are able to fine-tune their daily activity rhythms to habitat-specific human schedules, since these likely provide feeding opportunities. Behavioral plasticity may thus be an important driver of expansive population dynamics. Information on predictable relationships between daily activity patterns of gulls and human activities is therefore relevant to their population management.

## Introduction

Human impacts on the natural environment cause changes in the functioning of natural ecosystems worldwide, which has led a loss of biodiversity [1, 2]. In adition to the direct impacts on the structure and quality of natural habitats (e.g. habitat loss or degradation [3, 4]), human activities also are able to modify the spatio-temporal availability of trophic resources that some wildlife species are able to exploit efficiently [5]. Opportunistic wildlife is expected to respond to these changes by altering their distribution and activity patterns according to the availability of trophic resources [6, 7]. In fact, it has been described that routines of human activity within anthropogenic-dominated landscapes could schedule the behavior of the organisms living in these habitats [5].

In particular, humans may facilitate or hinder access to particular trophic resources at specific times of day, depending on their own movements and activity [8–10]. Accordingly, some species are able to adapt to the predictable dynamics in human-driven food availability, and are thus capable of taking advantage of highly anthropic scenarios [11, 12]. For many of these opportunistic species, behavioral adjustments represent the first response to altered environmental conditions [13, 14], serving as a foundation of their populations’ expansions in highly impacted areas [15, 16]. Thus, a better understanding of the ecological adaptations of opportunistic species in terms of shaping foraging activity rhythms on human activities is essential to managing the impacts of these activities [9, 17]. However, while previous research has largely focused on the study of spatial ecology of wildlife species [18, 19] and activity rhythms have been investigated extensively in marine environments [20], studies evaluating how those rhythms match the scheduling of human activities have been less common [17, 21, 22]. One example is how seabirds that consume fishery discards, change the use of marine environment and the type of foraging movements between the days depending upon working days of vessels [9, 23]. Similarly, human activity in particular terrestrial habitats associated to an increase in the number of people during holidays, also has been described as a source of variation in the spatial movements of raptors [24] and mammals [25].

The use of tracking devices has significantly improved the way we approach the movement of opportunistic species in response to both natural and human-induced environmental variability [19, 26, 27]. Tracking devices typically provide relevant information on the main foraging grounds of a particular organism, but can also inform on individuals’ decision-making on a near real-time basis over larger periods of time [19, 28]. This spatio-temporal data collection on individuals’ behavior, therefore, greatly benefits the study of activity patterns in relation to human activities [9, 23, 29].

In the present study, we used accurate GPS-tracking technology to precisely assess the foraging activity rhythms in relation to the habitat use in an opportunistic predator, the yellow-legged gull (*Larus michahellis*)*,* within a human-modified heterogeneous landscape. The yellow-legged gull, as other opportunistic gull species, is an suitable model to investigate behavioral adjustments in relation to daily patterns in human habitats use, as those animals are known to largely rely on anthropogenic food subsidies [5, 16]. Also, we evaluated the potential effect of human activity according to the type of day (working days, weekends and holiday days) on the 24-h foraging behavior of this opportunistic gull. Based on the previous knowledge for this and other closely-related species, we considered that scheduled human activities such as work timetables on weekdays and touristic or social activities on weekends would result in varying habitat-specific patterns according to feeding opportunities throughout the 24-h. We hypothesized that these contrasting patterns may influence the foraging activity rhythms both spatially and temporally [8, 9, 30]. Accordingly, we predicted that gulls would adjust their foraging strategies to varying human daily routines at certain habitats. In other words, we predicted that gulls would occur at particular times in those habitats where human activities generate the best feeding opportunities. Our study aims were therefore to provide, new valuable ecological information to understand the role of human activities in shaping foraging decisions in this paradigmatic opportunistic species model.

## Methods

### Study area

**Fig. 1:**
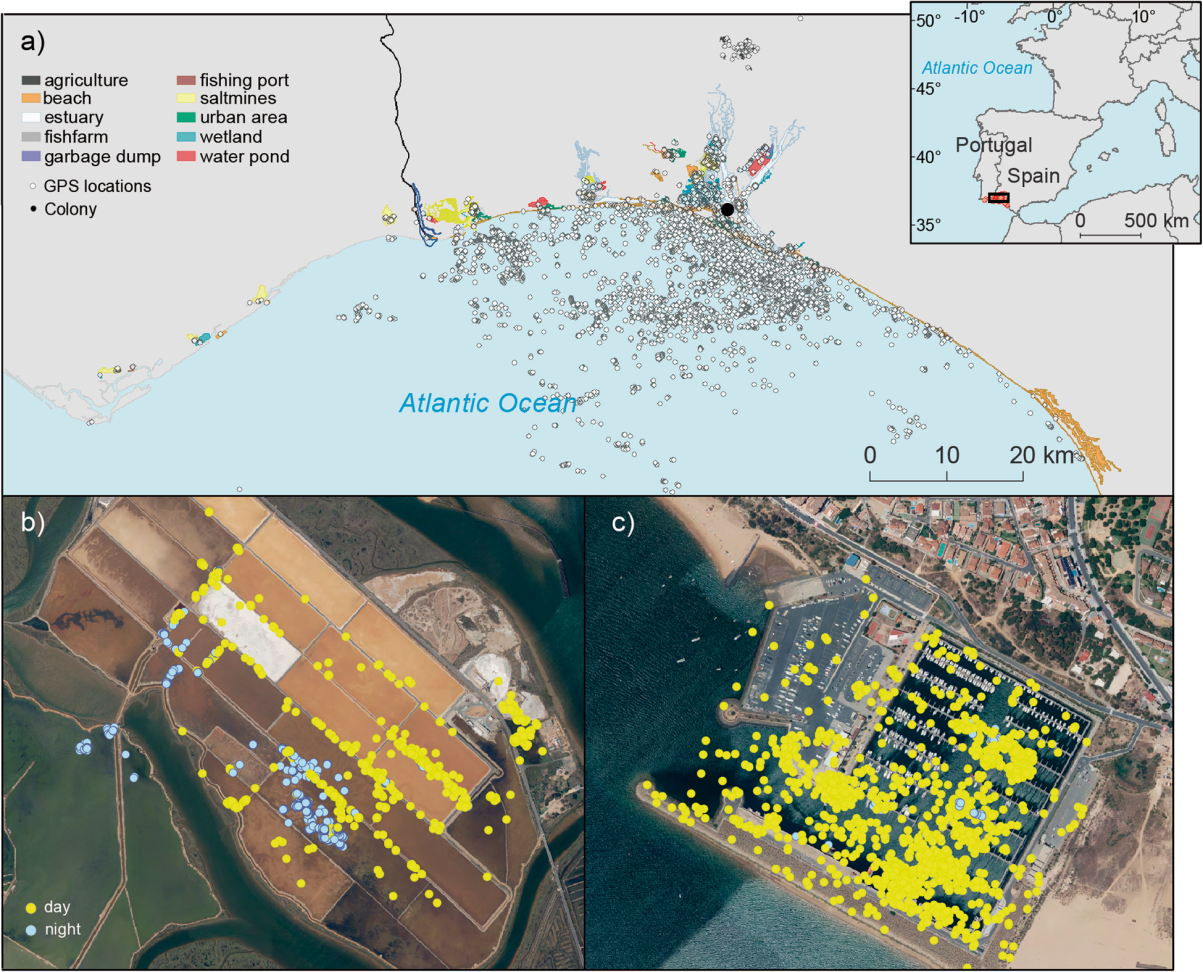
Study area showing (**a**) the spatial distribution of yellow-legged gull based on GPS-tracked information during the incubation period (14 May – 15 June 2015). **b** and **c** show two examples of yellow-legged gull locations in two different habitats (b = salt mines, individual 5257; c = fishing port, individual 5210). Yellow points display locations during the daytime, while blue points indicate nocturnal activity

The study was carried out at a breeding colony of around 300 pairs of yellow-legged gulls located in the natural protected Biosphere Reserve of Marismas del Odiel (37°13′N, 6°59′W, SW Iberian Peninsula, Gulf of Cadiz; Fig. 1) during the 2015 breeding period. Before investigating the activity patterns of each GPS-tracked gull, and following [31], we assigned the type of habitat associated with each GPS position by merging all filtered locations with high-resolution land cover information (SIOSE, Soil Information System of Spain, Junta de Andalucía; scale was 1: 10,000; last update 2013) and geographical references of waste dumps from the Spatial Reference Databases of Andalucía (DERA, last update 19/12/2018). This habitat classification was subsequently reviewed using the most recent satellite images offered by Google Earth V 7.1.2.2041 at a 0.5 m spatial resolution. Accordingly, habitats used by gulls were classified into eleven categories: estuary, wetland, beach, fishing port, salt mine, fish farm, water pond, agricultural area, sea area, urban area and garbage dump. Yellow-legged gulls were distributed over this heterogeneous and highly anthropogenic landscape, 60 km around their breeding colony [31].

### Tracking procedures

We deployed GPS-based UvA-BiTS loggers (www.UvA-BiTS.nl; [32]) on 18 breeding adult gulls, recording the positions of all individuals at 5-min intervals, continuously over the 24 h during all study period. Breeding gulls were captured using walk-in wire mesh traps over the nest. All GPS loggers were attached on the upper back using a Teflon wing harness [33]. The GPS logger and harness weighed less than 1.8% of the body mass of the birds, less than the 3% threshold suggested for seabirds [34].

GPS data from each logger were downloaded remotely through a local base station and automatically uploaded to the UvA-BiTS database [32]. To avoid possible biases associated with inter-individual differences in the number of days recorded by GPS tracking data, we focused our analyses on a period of time equivalently represented for all individuals: from 14 May to 15 June 2015 (incubation period for the species in this colony; [31]). We removed all locations inside the colony (using a radius of 500 m around tracked gulls’ nests) and all travelling locations (speed > 4 km·h^− 1^; [31]. The number of GPS locations ranged from 8200 to 9129 per individual, with a mean of 8644 ± 495 locations per individual (Table S1).

### Activity patterns analysis

We calculated the activity patterns in each habitat for each tracked gull as the total amount of time spent in each habitat per hour in relation to the entire tracking period. We then performed 24-h waveform analysis at a hourly basis in each habitat, to identify its temporal use over the 24-h for all tagged gulls. For this purpose, and to avoid biases due to differences in GPS acquisitions among individuals, first we standardized the activity data by the total number of daily GPS locations for each individual. Then, resulting time-series were partitioned into 24-h segments. Hourly values of all segments at a corresponding time were averaged together, also computing the standard deviation. The result was an averaged curve (i.e. the waveform) where the phase (as the significant increase in mean presence) was determined for each habitat by calculating the Midline Estimating Statistic of Rhythm (MESOR) [35]. MESOR is a threshold that can be computed by re-averaging all waveform values and is superimposed as a horizontal line on each waveform plot. Waveform values above the MESOR represented the phase indicating a significant period of occupation of an animal in an habitat over the 24 h cycle [36].

To evaluate the effect of the type of week day based on the scheduled human activity, waveform analyses were conducted separately for data gathered from Monday to Friday (workdays and weekends - Saturday and Sunday). For every case, the two average waveforms (weekdays vs. weekend) and the respective MESOR values were plotted simultaneously *per* habitat to check for possible differences in the activity patterns according to human activity. To compare the similarity in the activity patterns among habitats for weekdays and weekends, we constructed a similarity matrix based on Euclidian distances among different habitats. Euclidean distances were estimated based on the average number of GPS positions per hour and habitat for each type of day (weekday and weekend), following the Ward method [37] for grouping habitats using a clustering analysis. Approximately unbiased *p*-values were calculated by multiscale bootstrap resampling with 1000 replications. R software was used to conduct the cluster analysis.

### Results

**Fig. 2:**
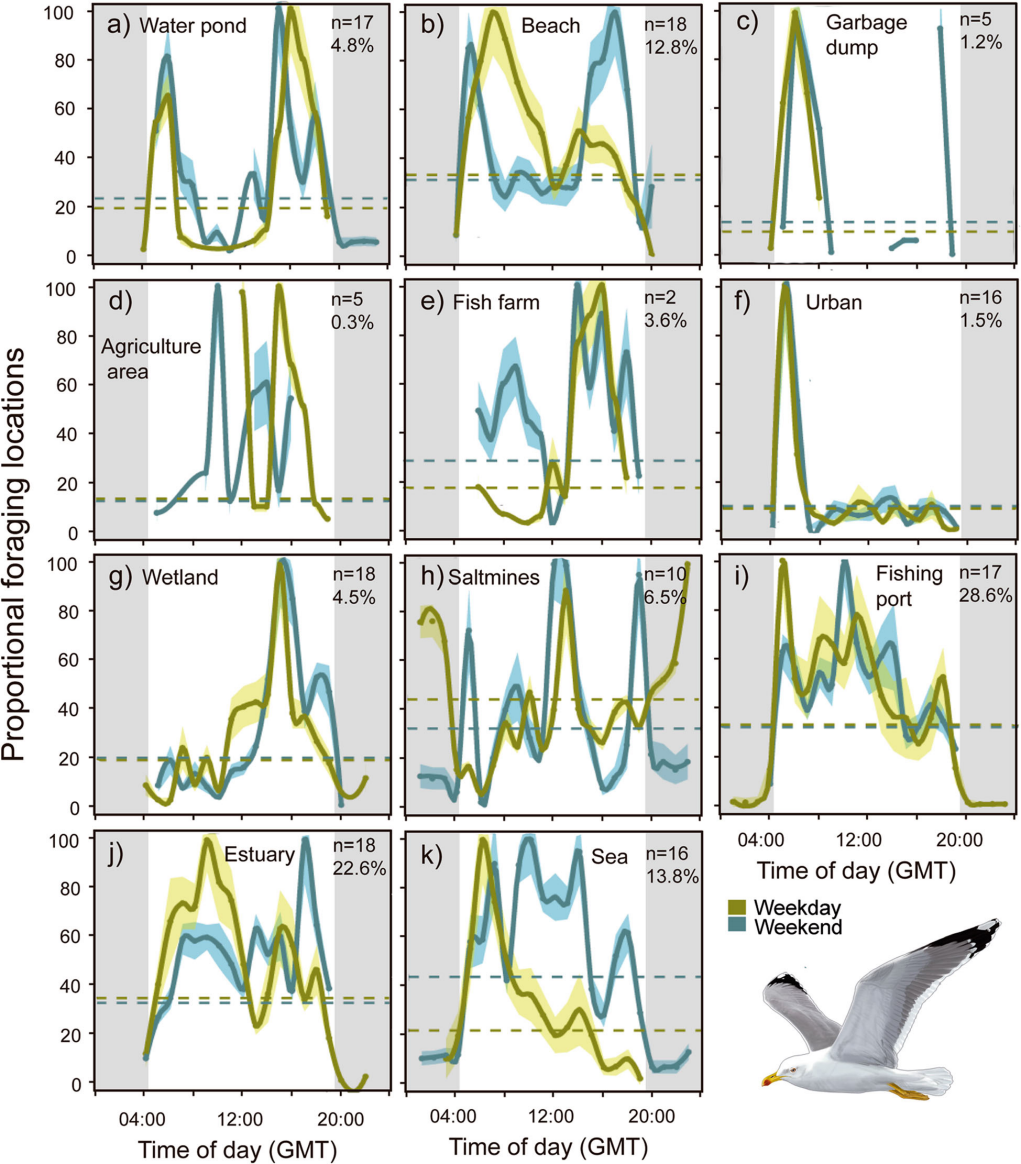
Population night-day cycle activity patterns (waveform) in each habitat used by yellow-legged gulls that were GPS-tracked during the breeding period in relation to the type of day (weekend or weekdays). The average number and the standard error (light yellow and blue areas) of GPS locations is shown. The horizontal dashed line represents the MESOR values. The number of individuals using each habitat and the importance in % of the use of each habitat is indicated in the upper right hand corner. The draw of yellow-legged gull was made by Martí Franch

Overall, waveform analysis showed a diurnal activity pattern between 4:00 and 19:00 h approximately (GMT) in all habitats, except for the salt mines, where activity peaks occurred at nighttime on weekdays (Fig. 2). In waveforms depicting diurnal activity, two peaks generally occured during the morning and evening hours, respectively, with a drop around noon. However, the presence and relative importance of these peaks (in terms of mean number of GPS locations) varied depending on the considered habitat (Fig. 2). On the contrary, other habitats such as ports, estuaries and the open sea showed a diurnal unimodal activity waveform (Fig. 2).

#### Weekend vs. weekday

**Fig. 3:**
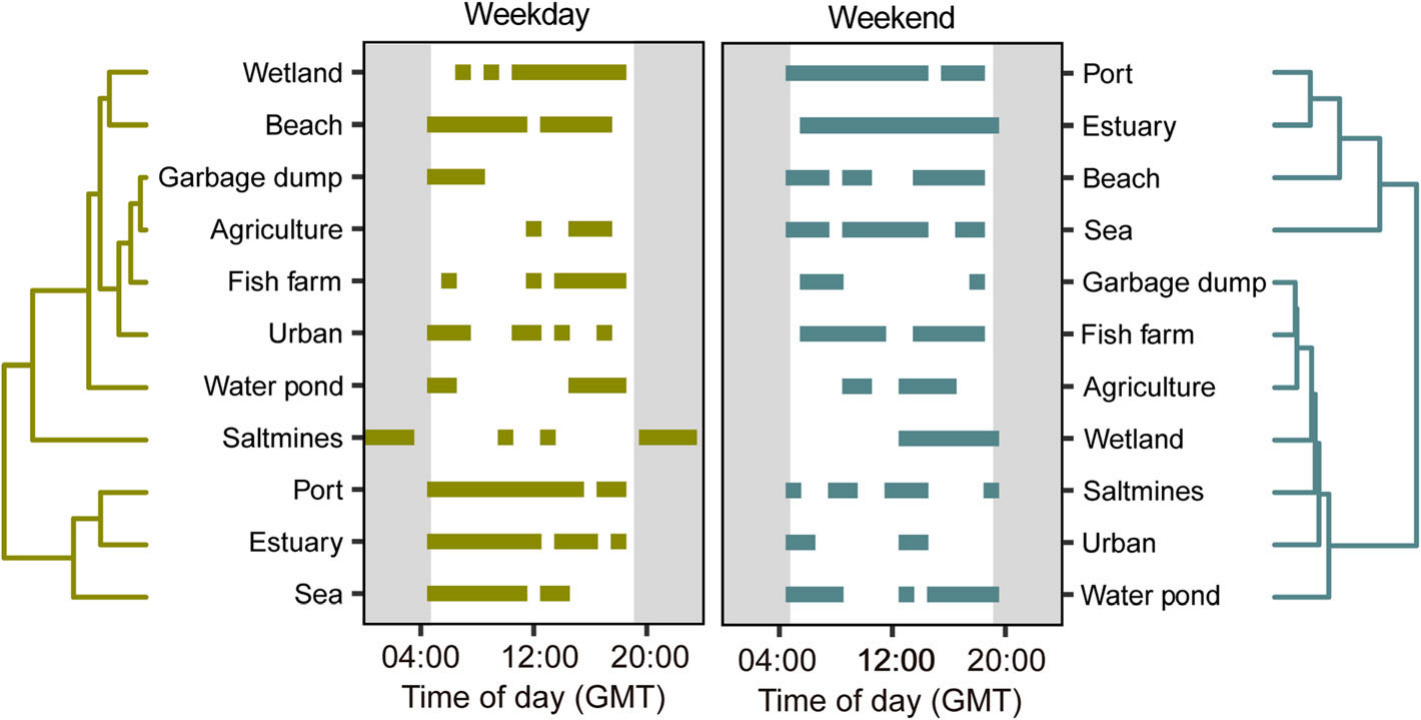
Dendrogram results showing the groupings of different habitats according to their night-day hourly activity in the sampled month for weekdays and weekends. The periods with a significant activity pattern according to the MESOR statistic by the cluster grouping are also shown for weekdays and weekends

Gulls structured their daily activity rhythms differently on weekdays and weekends, as indicated by the different classification of habitats based on habitat-specific activity patterns for tracked gulls (Fig. 3). All groups showed high significance (*p*-values ranged 0.74–0.99 for weekdays and 0.68–0.94 during weekends). Indeed, two main groups of habitat categories arose on weekends, including marine-related (port, estuary, beach, sea) and terrestrial habitats (garbage dumps, fish farms, agricultural land, wetland, salt mines, urban areas, water ponds). In contrast, no clear clustering was observed on weekdays. Water ponds, urban areas and wetlands were the only habitats showing similar patterns of use throughout the entire week (Fig. 2). In contrast, morning activity was significantly greater than evening activity on weekdays for beaches, garbage dumps, ports, estuaries and sea areas. In agricultural areas and fish farms, the peak of significant activity in the morning disappeared on weekdays, and in the case of salt mines, the pattern of nighttime activity disappeared on weekends. The clustering analysis of habitats based on activity similarity in terms of (i) hours with significant activity patterns and (ii) frequency of habitat use during these hours, grouped the 11 considered habitats into five categories with similar behavioral rhythms during a day-night cycle for weekdays and into two categories for weekends (Fig. 3). In particular, for weekdays, wetland and beach areas were grouped together, both with a high frequency of use during the afternoon and without any significant activity pattern around noon. The second category (garbage dumps, agricultural areas, fish farms, and urban areas) was grouped together for the terrestrial habitats due to the lower frequency of use. Water pond areas were classified in an isolated cluster due to the strict bimodal activity waveform. Salt mines were the only habitat with a significant activity pattern at night, and thereby were also classified in an isolated cluster. This habitat had two significant peaks of activity, during the night and at noon. Finally, ports, estuary areas and sea areas, which showed significant activity patterns throughout the day and over the whole week with decreasing frequencies of use towards the evening on weekdays, were grouped in the last cluster. On the other hand, the two groups of habitats resulting from the cluster analysis for weekends corresponded to the sea-land separation of habitats.

## Discussion

Human activity may modify spatiotemporal resource distribution, and therefore modulate spatiotemporal animal behavior as they take advantage of new feeding opportunities of anthropogenic origin [38, 39] or avoid human interactions [24, 40]. Accordingly, we provided quantitative evidence supporting the role of human activities and scheduled routines in shaping the foraging behavior and activity rhythms of an opportunistic and highly adaptable seabird, inhabiting a highly anthropogenic landscape. In particular, we reported daily behavior rhythms of yellow-legged gulls in the different habitats fitting within the different schedules of human activity. These results thus support the plasticity of the behavior of this gull species and their dependence on anthropogenic food subsidies [10, 41, 42].

Human activities can impact wildlife in highly diversified manners, including direct disturbances even resulting in direct mortality [43] resource facilitation [41]. All those influences are shaped by human routines as imposed by societal schedules [9, 23]. For instance, contrasting human activities between weekdays and weekends have been reported to affect the spatial distribution of species under the form of avoidance [24, 43] or facilitation of resources that would not be naturally accessible otherwise [23]. In this latter regard, fisheries, fish farms, livestock, crops and garbage dumps, may provide wildlife with a range of abundant and predictable food resources that have profoundly affected on its foraging behavior and associated activity rhythms, being this influence stronger for scavengers [5]. Opportunistic scavengers are capable of shaping their behavioral schedules and habitat use depending on human activities [44]. Our targeted gull species largely occur at highly anthropogenic areas and habitats [31, 41]. The targeted population inhabit a densely populated urban coastal zone [45], characterized by intense fishing and agricultural activities [46, 47]. Following our intial hypothesis, we provide evidence that individuals were also able to fine-tune their daily activity rhythms to day-specific human routines and schedules (weekdays vs. weekends) at these particular habitats, since these likely provided feeding opportunities for individuals.

Overall, the activity patterns of gulls in the marine-related habitats (port, estuary, and open sea) encompassed 65% of the foraging activity, with unimodal activity waveforms that peaked at mid-morning. According to previous studies, the yellow-legged gull mainly exploits marine preys [42] with some variation in dietary preferences among breeding colonies [48]. Pelagic and benthic coastal animals have a natural variability in their availability throughout the day due to the endogenous rhythms of physiology and behavior of coastal marine animals, such as vertical migrations associated with oceanographic processes [49]. However, availability of marine resources for seabirds may also be related to daily anthropic rhythms, mainly associated with fishing activities [44, 50]. Indeed, the mid-morning peak of activity in our tracked gulls within the marine habitat showed a clear match with fishing vessel arrivals to the ports and the moment when fishermen discard the non-commercial fish, suggesting that human activities can alter the species rhythmicity [9, 29]. Sea foraging activity was lower and more limited in time during weekdays, with maximum activity peaks occurring at specific hours and during much shorter time periods, thus suggesting exploitation of a highly predictable food resource in space and time, such as fishing discards. Contrastingly, gulls likely extended their foraging time at sea during weekends, to compensate for lower intake rates when no fishing discards are available [9]. These results contrast with previous patterns observed in Audouin’s gull (*Ichthyaetus audouinii*) that showed a shift towards an increasing use of terrestrial or freshwater habitats during weekends [9]. Our results indicate a more unaltered foraging behavior in gulls during weekends. This may also explain the observed differences in how these gulls structured their daily activity rhythms, with more clustered behaviors during weekends when certain human activities decrease, and hence, so does the abundance of related food subsidies.

Gulls’ activity patterns, mainly analyzed in relation to marine ecosystems and fishing activity [8], are far better understood as alternative “secondary” habitats (in terms of usage) frequented by gulls [30]. Bimodal activity patterns were generally seen in these “secondary” habitats, such as water ponds, fish farms, and beach area. These habitats, and hence their use by gulls, may be influenced by humans in different and divergent ways. Whereas in some habitats (e.g., salt mines, port, estuary and sea areas) the peak of maximum gull activity coincides with the times of greatest anthropic use as a likely response to enhanced food availability or facilitation, in other habitats (e.g., beach, garbage dumps, agricultural areas, fish farms and urban areas) these peaks occurred during the hours of less human influence, and hence, potential disturbance [30, 51]. An example of this is the activity, we detected on weekday nights at salt mines coinciding with the human working schedule in this habitat, suggesting that gulls may be feeding on associated resources. In contrast, gulls may be avoiding times of maximum human presence at particular habitats such, for example, the case of beach areas, where gulls are deterred by leisure activities from mid-morning to noon on weekends [51], but where they activity increase with respect to patterns observed on weekdays. Similarly, maximum peak activity in urban areas was observed during the early morning, when human activity is lower [30]. In agricultural areas gull activity was mainly observed in the mid-afternoon, when the weekday usually ends in those areas, and on weekends nearly throughout the day. Specifically, in the agricultural fields it has been observed that similar gulls take advantage of the newly plowed land to feed on earthworms and insects [52]. Similar phenomena have been observed in other species of shorebirds and gulls in areas on the southern coast of Spain, where the increase in recreational use limits the capacity of the site as a post-breeding stop-over area, and reduces the time that species spend on the consumption of prey in the presence of people on the beach [51]. The opposite case has been shown in several bird species, including gulls and other waterbirds using urban environments and showing habituation to human disturbances [53].

## Conclusions

In conclusion, our study reveals a link of anthropic activity patterns to the schedule of foraging behavior in an opportunistic predator living in a heterogeneous human-modified landscape. The anthropogenic scheduled activity is linked to gulls’ temporal exploitation of the main feeding habitats, while resting activities are decoupled from anthropic rhythms. Our work highlights the importance of fine-scale habitat use studies, both temporal and spatial, to explore adaptive individual plasticity in habitat use.

## Supplementary information

**Additional file 1: Table S1.** Total number of filtered GPS positions (GPS) recorded by 18 yellow-legged gulls (*Larus michahellis*) during one month of the 2015 breeding season in Odiel (southeaster Iberian Peninsula, Spain).

## Data Availability

Datasets available in the UvaBits repository http://www.uva-bits.nl/virtual-lab/, Access to these data is available from the corresponding author on reasonable request.
