# Supplementary material for: Behavioral rhythms of an opportunistic predator living in anthropogenic landscapes
Source: Mov Ecol. 2020 Apr 24;8:17. doi: 10.1186/s40462-020-00205-x (PMC7183138; doi:10.1186/s40462-020-00205-x)
Supplement: Supplementary file 1 — Additional file 1: Table S1. Total number of filtered GPS positions (GPS) recorded by 18 yellow-legged gulls (Larus michahellis) during one month of the 2015 breeding season in Odiel (southeaster Iberian Peninsula, Spain). [file 40462_2020_205_MOESM1_ESM.docx]

| **Table S1**. Total number of filtered GPS positions (GPS) recorded by 18 yellow-legged gulls (*Larus michahellis*) during one month of the 2015 breeding season in Odiel (southeaster Iberian Peninsula, Spain) | |
| --- | --- |
| Individual | GPS positions |
| 5208 | 747 |
| 5210 | 1857 |
| 5227 | 532 |
| 5229 | 1009 |
| 5238 | 1580 |
| 5241 | 662 |
| 5242 | 730 |
| 5244 | 1164 |
| 5246 | 502 |
| 5248 | 407 |
| 5250 | 501 |
| 5255 | 3955 |
| 5256 | 1466 |
| 5257 | 1386 |
| 5259 | 1188 |
| 5260 | 699 |
| 5264 | 1724 |
| 5268 | 1584 |
